# Performance Improvement of AlN Crystal Quality Grown on Patterned Si(111) Substrate for Deep UV-LED Applications

**DOI:** 10.1038/srep35681

**Published:** 2016-11-07

**Authors:** Binh Tinh Tran, Noritoshi Maeda, Masafumi Jo, Daishi Inoue, Tomoka Kikitsu, Hideki Hirayama

**Affiliations:** 1 RIKEN Center for Advanced Photonics, 2-1 Hirosawa, Wako, Saitama 351-0198, Japan; 2 Quantum Optodevice Laboratory, RIKEN, 2-1 Hirosawa, Wako, Saitama 351-0198, Japan; 3 Center for Emergent Matter Science, RIKEN, 2-1 Hirosawa, Wako, Saitama 351-0198, Japan

## Abstract

An AlN template layer is required for growth of AlGaN-based deep ultraviolet light-emitting diodes (UV-LEDs). However, the crystal quality of AlN templates grown on both flat and patterned Si substrates has so far been insufficient for replacing templates grown on sapphire substrates. In this work, we grew a high-quality AlN template on 2 in. micro-circle-patterned Si substrate (mPSiS) with two different sizes and shapes through controlling the bias power of inductively coupled plasma (ICP) etching. The experimental results showed that the best AlN template was obtained on a large pattern size with a bow-angle shape and the template had X-ray rocking curves with full widths at half-maximum of 620 and 1141 arcsec for the (002) and (102) reflection planes. The threading dislocation density near surface of AlN template through transmission electron microscopy (TEM) estimation was in the order of 10^7^ cm^−2^, which is the lowest dislocation density reported for a Si substrate to our knowledge. A strong single electroluminescence (EL) peak was also obtained for an AlGaN-based deep UV-LED grown on this template, means that it can be used for further developing high-efficiency deep UV-LEDs.

Epitaxial growth of the group III-nitride semiconductors on either Si or sapphire substrates has attracted great attention because these materials have excellent properties and many applications. AlN is an important material of group III-nitride semiconductor because it is required for developing high-power electronic and optoelectronic devices, such as radiofrequency filters, high electron mobility transistors, microelectromechanical systems^1^,^2^,^3^,^4^. In particular AlN is needed for AlGaN-based deep UV-LED applications^5^,^6^. AlGaN-based deep UV-LEDs require that a thick, high-quality AlN template be grown on the Si substrate before the other epitaxial layers can be grown. The AlN template allows emission of a very short wavelength (~210 nm), which is the most important property for deep UV-LED applications. In addition, the Si substrate has many advantages: it can be removed by chemical treatment to allow back illumination, it prevents the generation and reabsorption of UV light by backside emission^7^,^8^,^9^, it is inexpensive, and large sizes are available. However, Si-based AlN substrates for deep UV-LEDs are less widely used than sapphire substrates owing to the large lattice mismatch (~23.4%) and thermal expansion coefficient between AlN and Si compared with the sapphire substrate. Thus, replacing the conventional sapphire substrate with Si is a major challenge preventing commercialization of AlN substrates.

Many methods have been used to improve AlN templates grown on either sapphire or Si substrates, such as native bulk AlN substrates, migration-enhanced metal organic chemical vapor deposition (MOCVD) growth, pulsed-flow multilayer AlN growth, growth mode modification, and high-temperature growth to grow AlN templates on stripe patterned AlN/Si or AlN/sapphire substrates^9^,^10^,^11^,^12^. The methods suppress the main problems arising from the differences between AlN and substrate during growth including low growth rate and lattice mismatch, which prevent the improvement of AlN templates on substrates and cause high dislocation density and crack initialing stress. For the growth of AlN on Si substrate, the presence of the native oxide layer on the Si substrate leads to low coherence between the AlN template and the Si substrate, making it difficult to obtain a smooth AlN surface^13^,^14^. The crystal quality of AlN grown on flat or patterned Si substrates is still low compared with the AlN/sapphire substrate^8^,^9^,^15^,^16^,^17^,^18^.

In this letter, we report the fabrication of high-density mPSiS with different pattern sizes and shapes for comparison for further growth of thick AlN template. To grow thick AlN templates directly on the substrates, we use NH_3_ pulsed-flow multilayer AlN growth and ELO (epitaxial lateral overgrowth) techniques in a MOCVD reactor. Especially, the mPSiS fabrication is simpler, faster, and more effective than other methods, and the growth conditions are optimized with slight differences from previous report^19^. The AlN crystal quality effected by pattern sizes and shapes are mainly studied and evaluated by using X-ray diffraction (XRD), atomic force microscopy (AFM), scanning electron microscopy (SEM), (TEM), and etch pit density (EPD) to determine the crystal quality, surface roughness, thickness, and dislocation density, respectively. Finally, an AlGaN-based deep UV-LED was also grown on that template, carried out the EL measurement and compared with another AlGaN-based deep UV-LED which grown on a lower crystal quality AlN template to prove that the template is useful and it can be used to develop deep UV-LED devices with high quantum efficiency.

## Results and Discussion

**Figure 1:**
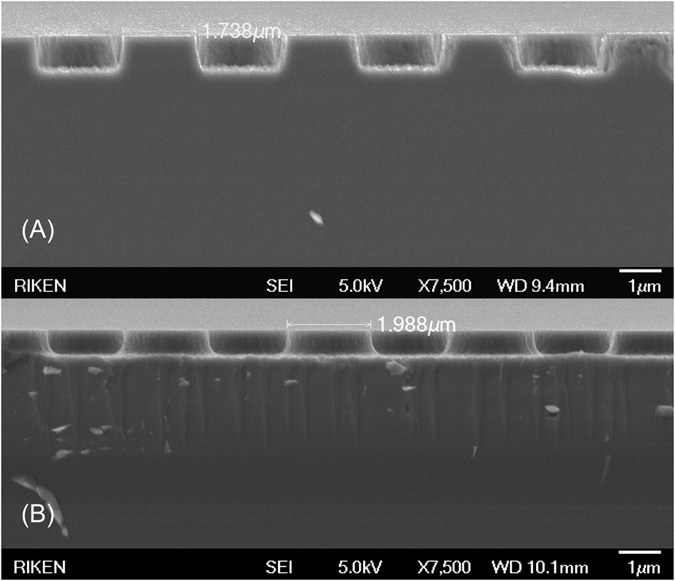
Shows cross-sectional SEM images of (**A**) mPSiS-A (~1.7 μm in diameter and 1 μm deep, obtained with 10 W bias and 50 min ICP etching) and (**B**) mPSiS-B (~2.0 μm in diameter and 0.6 μm deep, obtained with 5 W bias and 40 min ICP etching).

**Figure 2:**
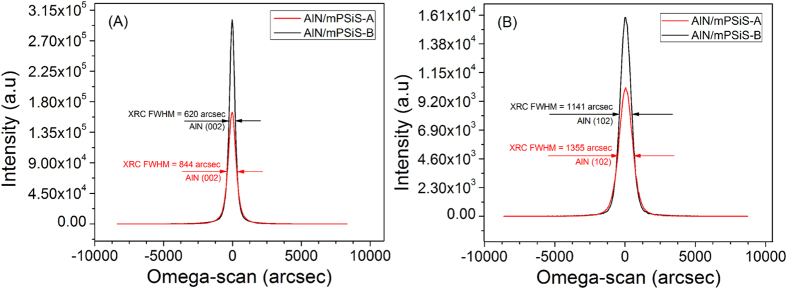
XRC spectra for (**A**) symmetrical and (**B**) asymmetrical planes of about 8 μm AlN/mPSiS-A (Sample-A) and AlN/mPSiS-B (Sample-B). The crystallinity is 25% better for the symmetrical planes and 15% better for asymmetrical planes in AlN/mPSiS-B compared with AlN/mPSiS-A.

The SEM images of mPSiS etched with different bias powers showed different pattern sizes and shapes (Fig. 1). For the mPSiS etched with a bias of 10 W (mPSiS-A), the angle of the pattern sidewall and the ground was nearly perpendicular, although mPSiS was etched with a bias of 5 W (mPSiS-B), the angle changed to 45°-bow and the pattern sizes were also larger than those for mPSiS-A. We found that by controlling the ICP bias power, it can suppress the wear of the mask, which usually affects the results. The two types of patterned-substrates then loaded into the MOCVD reactor to grow thick AlN templates. Figure 2 shows the XRD rocking curves (XRC) of the AlN templates grown on these substrates with a similar thickness of about 8 μm. The FWHMs of the AlN template grown on mPSiS-B (Sample-B) were about 25% lower (i.e better) than for those grown on mPSiS-A (Sample-A) for the symmetrical reflection planes (Fig. 2(A)) and 15% for the asymmetrical (Fig. 2(B)) reflection planes. Thus, the AlN template grown on mPSiS-A had a FWHM of 844 (002) and 1355 (102) arcsec, and that grown on Sample-B was a FWHM of 620 (002) and 1141 (102) arcsec. This result indicates that the patterns really affected the crystallinity of the AlN template and are the closest values to those for AlN grown on a sapphire substrate.

**Figure 3:**
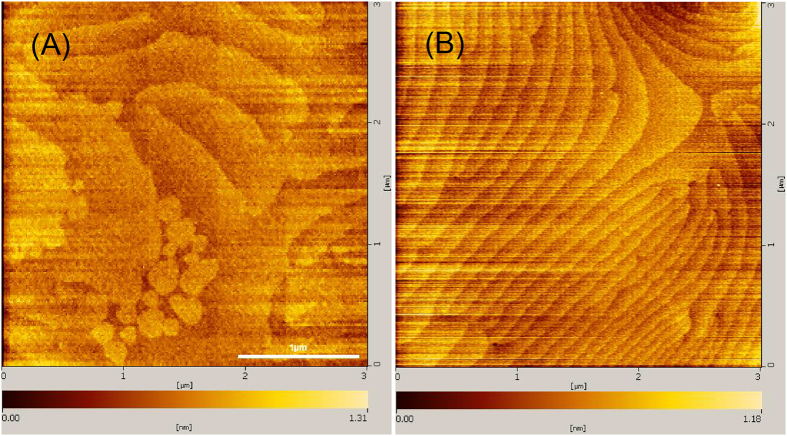
(**A**) AFM images of the surface roughness of Sample-A and (**B**) Sample-B.

AFM images showed the surface roughness was about 3.5 nm for Sample-A and 1.6 nm for Sample-B (Fig. 3). Moreover, on the surface of Sample-B, atomic steps with high, well-defined terraces were clearly observed, indicating that high coalescence was obtained. This observation shows that the threading dislocation density on the surface was very low (confirm by TEM measurement later) since it was sharply bent at the bottom and could not reach the surface as it has ever happened in Sample-A^19^ with a thick AlN layer.

**Figure 4:**
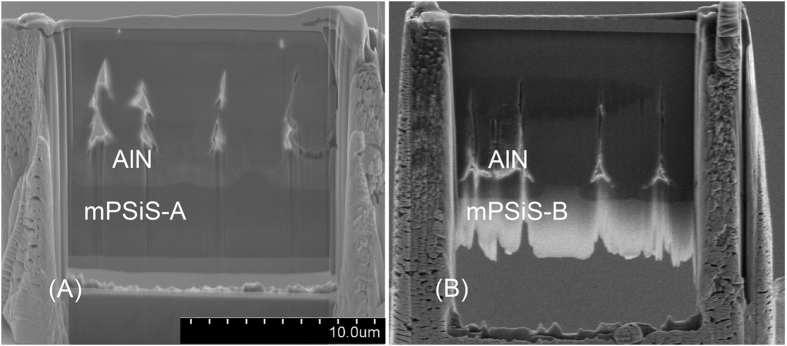
Cross-sectional SEM images of (**A**) Sample-A and (**B**) Sample-B after focused ion beam processing.

Figure 4(A,B) compare the cross-sectional SEM images of the morphologies of Sample-A and Sample-B show the thickness of Sample-B was slightly thicker than Sample-A. The coalescence layer was established sooner in Sample-B than in Sample-A, about 3 μm from the bottom, and the perfectly coalesced thickness was about 3 μm thick on the top. The shape and large size of mPSiS-B may have resulted in the AlN template combination happening faster than the growth of the AlN template on mPSiS-A, and thus the XRD FWHM of Sample-B was better than that of Sample-A.

**Figure 5:**
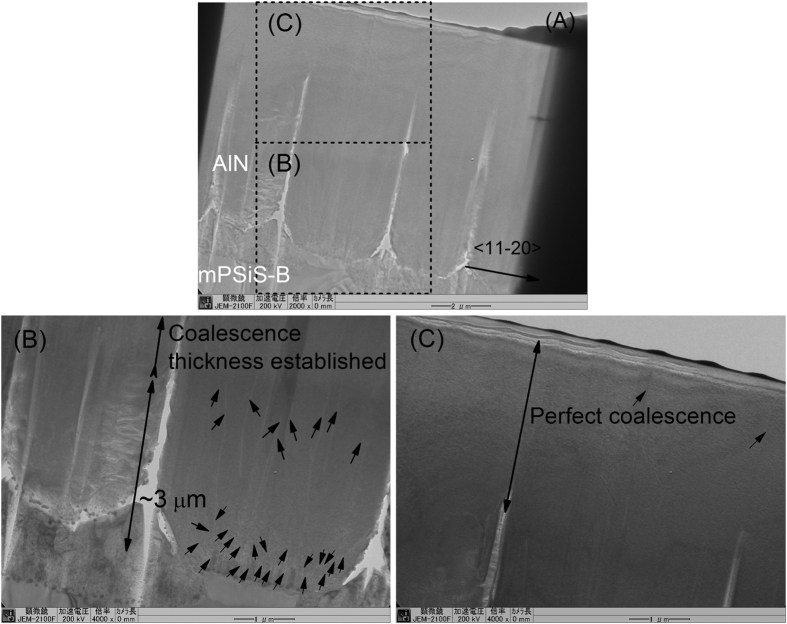
(**A**) Cross-sectional TEM images of Sample-B taken under two-beam conditions along [11-20], and (**B**), (**C**) magnifications of the image in (**A**). There are some dislocations at the bottom region (indicated by arrows), fewer in the middle of the template layer, and almost none in the top region.

TEM and EPD are the most common techniques for monitoring the threading dislocation density^20^. To measure the crystal quality of AlN grown on these substrates, TEM and EPD were used to observe the threading dislocation densities in the cross-sectional and plane view areas. The bright-field cross-sectional TEM images were obtained with g = <11-20> (TEM JEOL 2100F). The cross-sectional images showed some threading dislocations in the large area at the bottom of the template (Fig. 5(A)) and are clearer in the magnified images (Fig. 5(B,C)). ELO growth resulted in most of the threading dislocations being distributed at the bottom, around the patterns, and the dislocations were bent toward the sidewall of voids, terminated at the sidewalls, and could not reach the AlN surface (Fig. 5(C)). AlN/mPSiS-B showed an excellent coalescence thickness with no gaps. The dislocation densities estimated from the TEM image in the perfect coalescence region (near the surface) of AlN/mPSiS-B were approximately 5 × 10^7^ cm^−2^ (screw) and 7.5 × 10^7^ cm^−2^ (edge) and in the order of 10^8^–10^9^ cm^−2^ in the middle to bottom. These values were low compared with the values for Sample-A of 1.5 × 10^8^ cm^−2^ (screw) and 3.7 × 10^8^ cm^−2^ (edge)^19^.

**Figure 6:**
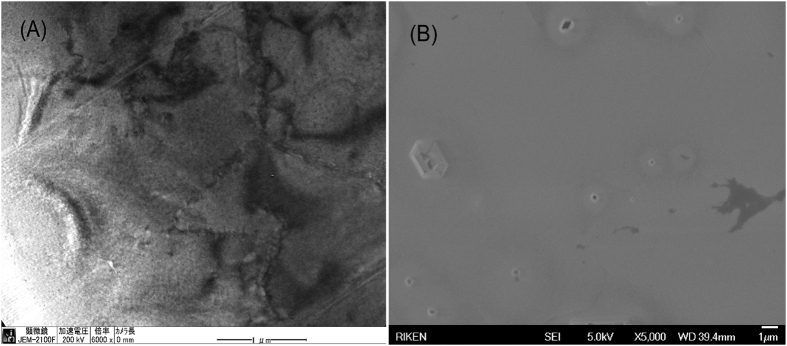
Plan-view images of Sample-B taken by (**A**) TEM and (**B**) EPD/SEM.

The threading dislocation density on the AlN surface was determined by TEM and EPD. For EPD, we soaked the AlN sample in KOH solution at 75 °C for 50 sec. A thin conductive layer was deposited on the etched surface before it was imaged by SEM to calculate the dislocation density (Fig. 6).

The plan-view TEM image of Sample-B (Fig. 6(A)) shows dislocations, although they were unclear and could not be identified as screw, edge, or mixed dislocations, although the plan-view TEM images taken with g = <11–20> showed that most were edge dislocations. Therefore, EPD was performed and a scanned area of about 3 × 10^6^ cm^−2^ was obtained from the SEM measurements (Fig. 6(B)). The threading dislocation density calculated from the EPD results may be lower than that calculated from TEM results (Fig. 6(A)) because of the differences in observation techniques. TEM has a very high spatial resolution that can detect nanoscale objects, whereas SEM is a microscale technique and cannot detect some dislocations. In addition, some threading dislocations also disappeared before they could reach the AlN surface as mentioned above. Generally, these dislocation densities indicated that mPSiS-B is an excellent template for developing high-efficiency deep UV-LEDs.

**Figure 7:**
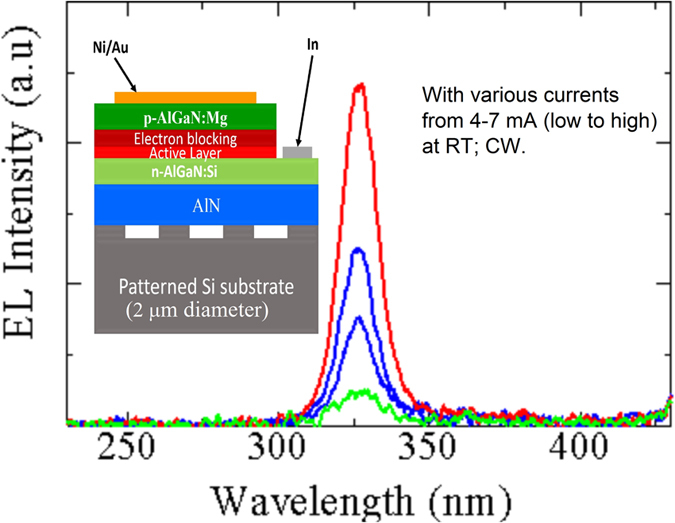
An AlGaN-based deep UV-LED has been fabricated (inset) and obtained a sharp single EL peak at 325 nm.

To demonstrate that mPSiS-B allowed further growth of deep UV-LED devices with high quantum efficiency, an AlGaN-based deep UV-LED has been fabricated device as shown in the inset of Fig. 7. The deep UV-LED structure was grown at 1200 °C for all layer, except the p-AlGaN was grown at 1150 °C. Figure 7 shows the EL spectrum of AlGaN-based deep UV-LED grown on this AlN template. The EL measurement was carried out at room temperature (RT) under CW operation. A single EL peak spectrum at 325 nm were clearly observed for the device. This result indicates that a high crystal quality AlN template can be used to improve the performance of a deep UV-LED and obtain a single sharp peak at 325 nm, which is shorter than the challenge wavelength at 350 nm since it can reduce the threading dislocation in upper layers.

## Conclusions

By combination of our previous growth conditions with slightly optimization and a new pattern fabrication, we have successful growth of a very high AlN template on Si substrate which can be compared with that grown on a sapphire substrate. A perfect micro-circle patterned Si substrate (2 μm diameter) identical to the SiO_2_ mask diameter with no pattern edge wear by controlling the ICP bias during etching and optimizing the other conditions was fabricated. The pattern shape was also important in establishing the coalescence thickness faster in mPSiS-B than in mPSiS-A. In addition, the thickness of the SiO_2_ mask was important for etching. We could not obtain the full 2 μm diameter pattern if the mask was thin and it was difficult to obtain a sufficiently high etch depth for the pattern if the mask was thick. The optimum SiO_2_ mask thickness was identified as 250 nm, although it also depended on the etching conditions such as gas flow. Overall, the AlN template could be directly grown on the patterned Si substrate and had an XRD FWHM of 620 and 1141 arcsec for the (002) and (102) reflection planes, respectively, and the near surface dislocation densities in the order of 10^7^/cm^2^ was also obtained. Additionally, we grew two AlGaN-based deep UV-LEDs on the high and low crystal quality AlN templates that a strong single EL peak has been obtained for a deep UV-LED grown on the better AlN template, confirming the AlN template grown on our patterned Si can be used for further growth of deep UV-LED devices with high quantum efficiency.

## Methods

Trimethylaluminum (TMAl) and NH_3_ were used as sources for Al and N, respectively, and N_2_ was used as carrier gas. The pattern fabrication have been reported elsewhere^19^, but the details of steps are as follows. Before pattern fabrication, the Si substrates were treated with a buffered oxide etchant, then rinsed with deionized water for 5 min to remove the surface oxide layer, and cleaned at 100 °C for 10 min in an oven. A ~250-nm-thick layer of SiO_2_ was deposited by CVD. The substrate was coated with hexamethyldisilazane at a rotation speed of 3000 rpm for 60 sec, and then baked at 80 °C for 30 sec. The photoresist (AZ5412) spin-coated onto the substrate at a rotation speed of 3000 rpm for 60 sec and baked at 80 °C for 30 sec. Standard lithography was performed for 3 sec and the substrate was baked at 120 °C for 3.5 min. Next, standard lithography was performed for 30 sec and the substrate was immersed in chlorobenzene for 2 min. The substrates were kept in a dry place for 60 min and were developed for about 7 min.

The substrate was etched by ICP using CF_4_ (20 sccm) for 2 min 35 sec at a bias of 100 W and power of 100 W, and then with CF_4_ (20 sccm) and O_2_ (3 sccm) at a power of 100 W. The substrate was soaked in acetone for 15 min to clean the surface of the photoresist, and then soaked in buffered hydrofluoric acid for 7 min to remove the SiO_2_ and obtain the mPSiS.

The total thickness of each AlN template included five AlN layers is about 8 μm. Where, the first, second, and fourth layers were grown by the NH_3_ pulsed-flow multilayer AlN growth technique. The TMAl and NH_3_ flows for these layers were 10 sccm, and the first AlN layers were grown at a pressure of 200 Torr and 76 Torr for other layers. The ELO technique was used for the third and fifth AlN layers with a flow of 35 sccm for TMAl and 5 sccm for NH_3_. All the samples were grown at 1380 °C for a total time of 149 min.

Different pattern sizes and shapes were obtained by etching the Si substrates with different ICP bias powers. For a pattern size of 1.7 μm, we used a bias power of 10 W and 50 min etching and reduced the power to 5 W and 40 min etching to obtain a pattern size of 2.0 μm (Fig. 1). The AlN templates grown on the pattern sizes of 1.7 and 2.0 μm are called Sample-A and Sample-B, respectively. For the growth, we reduced the growth temperature from 1390 to 1380 °C and increased the NH_3_ flow from 5 to 6 sccm for the third and fifth AlN layers^19^ to get a smooth AlN surface.

## Additional Information

**How to cite this article**: Tran, B. T. *et al*. Performance Improvement of AlN Crystal Quality Grown on Patterned Si(111) Substrate for Deep UV-LED Applications. *Sci. Rep.* **6**, 35681; doi: 10.1038/srep35681 (2016).

**Publisher’s note**: Springer Nature remains neutral with regard to jurisdictional claims in published maps and institutional affiliations.
